# Supplementary material for: Systematic mediation and interaction analyses of kidney function genetic loci in a general population study
Source: PLoS One. 2025 Jun 24;20(6):e0323057. doi: 10.1371/journal.pone.0323057 (PMC12186986; doi:10.1371/journal.pone.0323057)

Table of Contents

[Supplementary methods 2](#_Toc192720316)

[Supplementary Figure S1. Cumulative variance of 6337 variants over 147 loci explained by principal components in the CHRIS study. 3](#_Toc192720317)

[Supplementary Figure S2. Linear association models: principal component adjustment versus linear mixed modeling in EMMAX. 4](#_Toc192720318)

[Supplementary Figure S3. Quantile-quantile (QQ) plot of the P-values from the GWAS of ln(eGFRcrea) in the CHRIS study. 6](#_Toc192720319)

[Supplementary Figure S4. Regional association plots for the 11 significant loci. 7](#_Toc192720320)

[Supplementary Figure S5. Comparison of effect size and standard error ratio for the variants in the model regressing ln(eGFRcrea) on age, sex, FT3 (or FT4), genetic variants with and without adjusting for municipality. 13](#_Toc192720321)

[Supplementary Figure S6. Genetic overlap between CHRIS and 1000 Genomes project samples, showing that the CHRIS study matches the European ancestry. 15](#_Toc192720322)

# ****Supplementary methods****

***Quantile normalization of the 70 health traits used in the mediation analysis***

**We used** *normalize2Reference* function in the caret R package version 6.0-34 (the function is available in the later versions here: https://github.com/topepo/caret/tree/master/deprecated). Each trait was measured partially with an older method and partially with a newer, most recent method. The observations from the newer **method (method = 1) were set as the reference to derive the quantiles of the trait distribution on which to standardize the observations from the older method (method = 0). We then replaced the observation from the older method with the standardized ones and included together with the values from the newer method.**

**Notation:**

- **trait**: the quantitative variable to which quantile normalization was applied;
- **method:** the newer **method for measuring the trait (1); the older method (0);**
- **df: dataframe; the data in which the trait of interest is available**

library(caret)

quantile_norm <- normalize2Reference(

data = df[df$method == 0, "trait"],

refData = quantile(

df[df$method == 1, "trait"],

probs = seq(0, 1, length.out = length(df[df$method == 0, "trait"])),

na.rm = TRUE,

names = TRUE,

type = 7,

digits = 7),

ties = TRUE)

# Creating a new variable for the quantile normalized trait

df$trait_std <- df$trait

# Replacing non-transformed values with the quantile-normalized values

df[df$method == 0, "trait_std"] <- quantile_norm

# ****Supplementary Figure S1.** Cumulative variance of 6337 variants over 147 loci explained by principal components in the CHRIS study.**

Adjusting for 147 independent tests covers 98% of the cumulative variance (red dot).


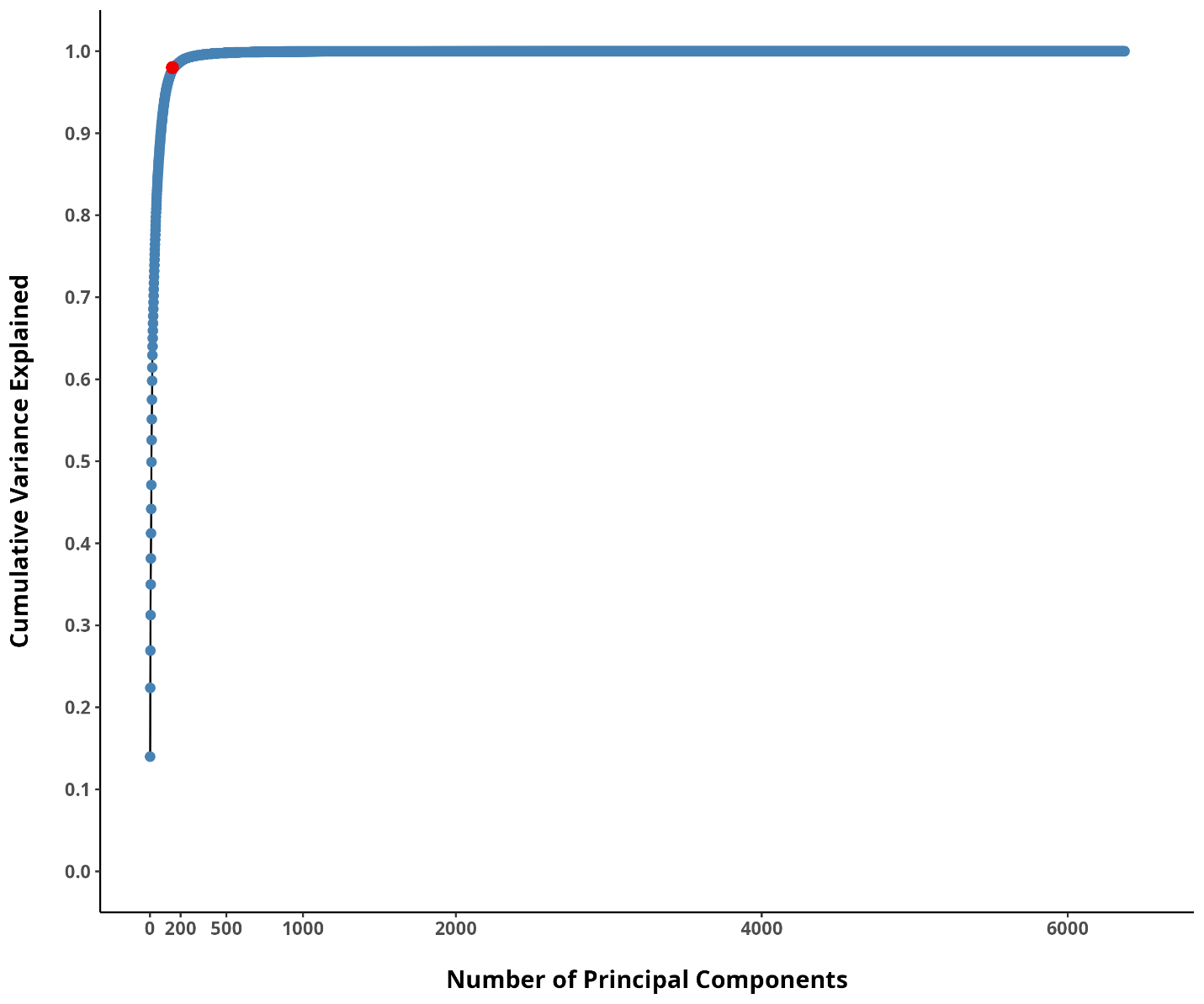


# ****Supplementary Figure S2.** Linear association models: principal component adjustment versus linear mixed modeling in EMMAX.**

We observed substantial equivalence between kinship-adjusted linear mixed models obtained with EMMAX and PC-adjusted models.


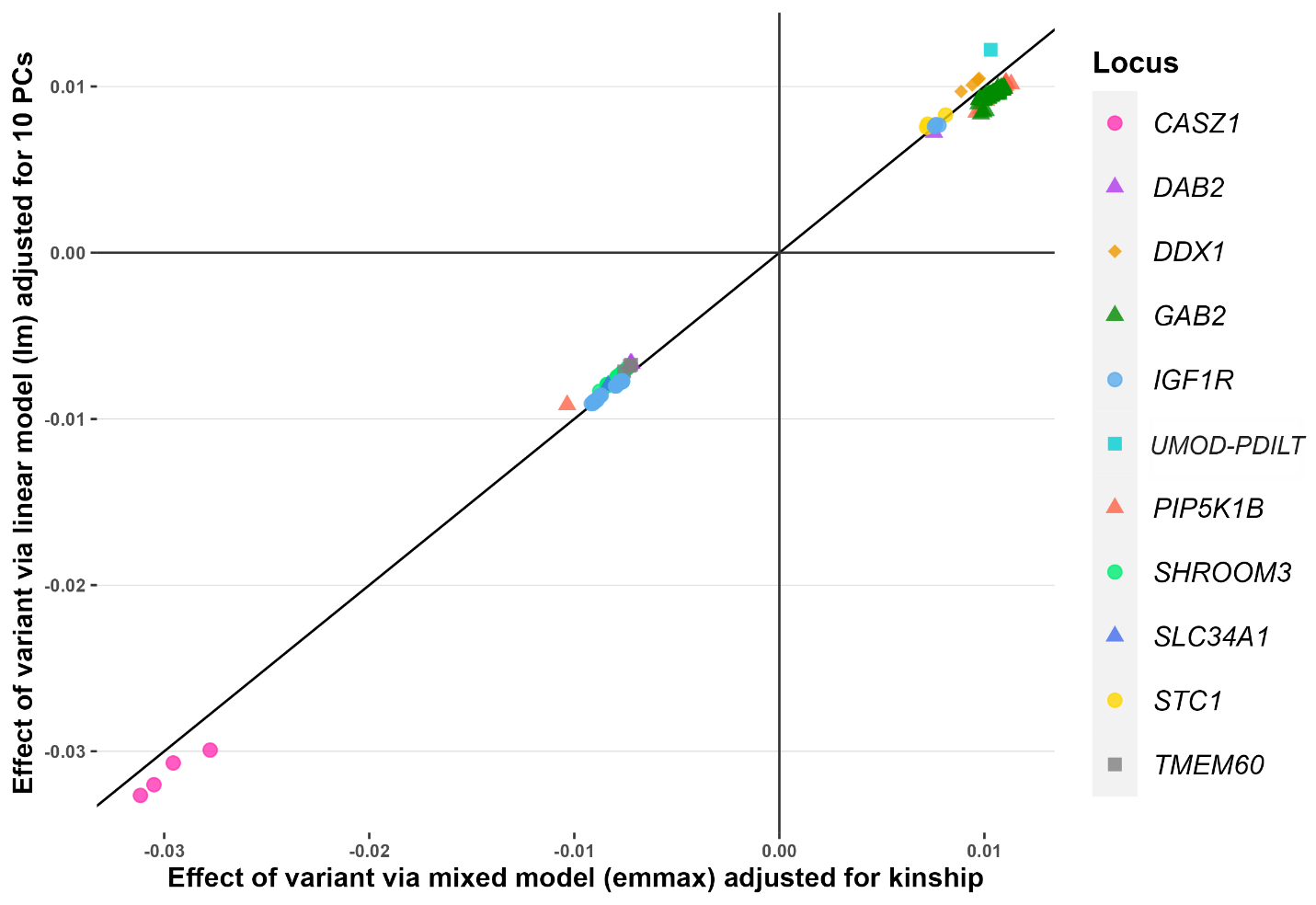


# ****Supplementary Figure S3.** Quantile-quantile (QQ) plot of the P-values from the GWAS of ln(eGFRcrea) in the CHRIS study.**


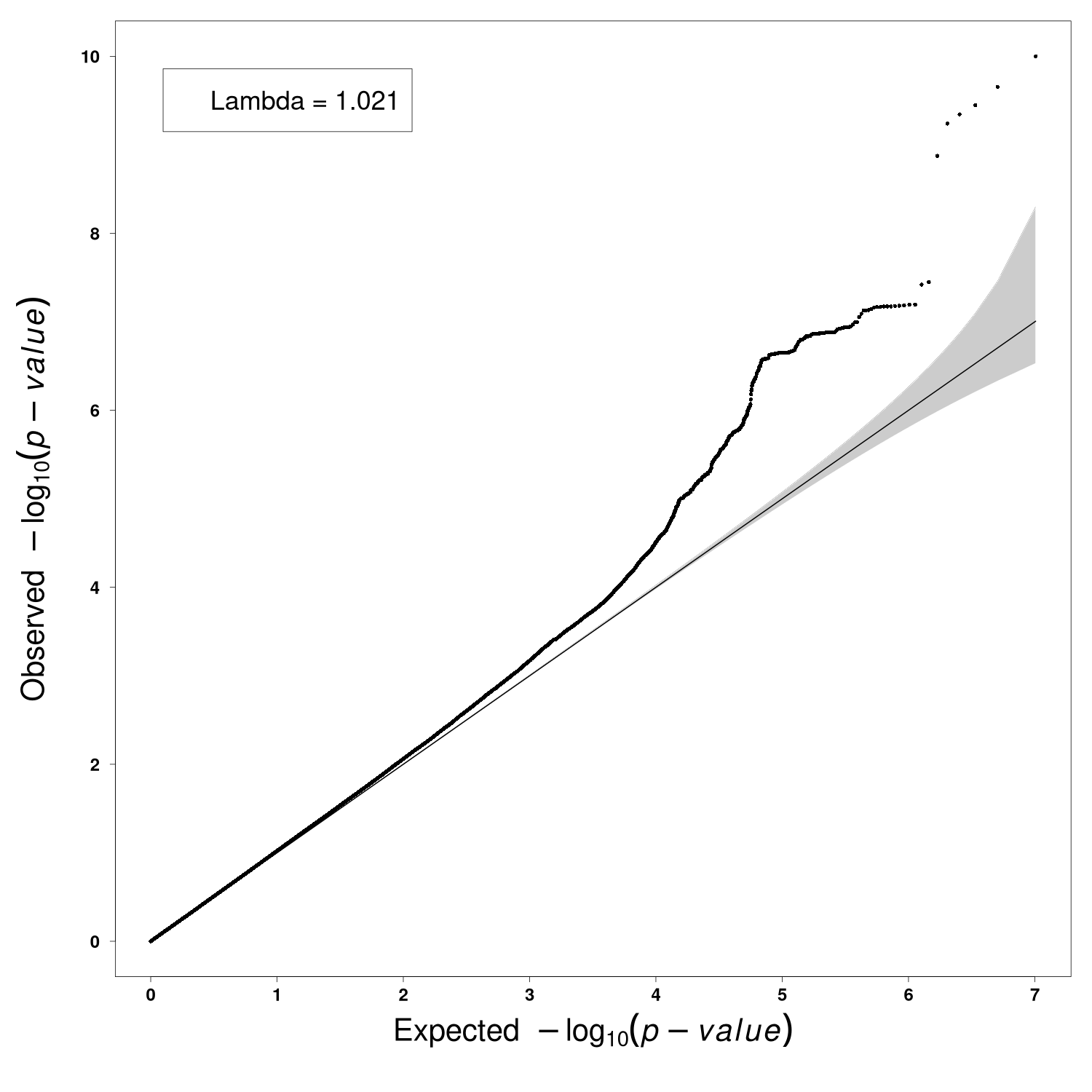


# Supplementary Figure S4. Regional association plots for the 11 significant loci.

Highlighted in purple is the most associated SNP in the CKDGen GWAS meta-analysis. All SNP positions are referred to the NCBI Build 38. Plots were generated with LocusZoom version 1.4 (Pruim RJ, et al., Bioinformatics, 2010. 26(18): 2336-7).

* indicates cases where not the CKDGen lead SNP but a proxy of it was identified in the CHRIS study.

Top CHRIS SNP: rs74748843 (1:10,670,853) at ***CASZ1***


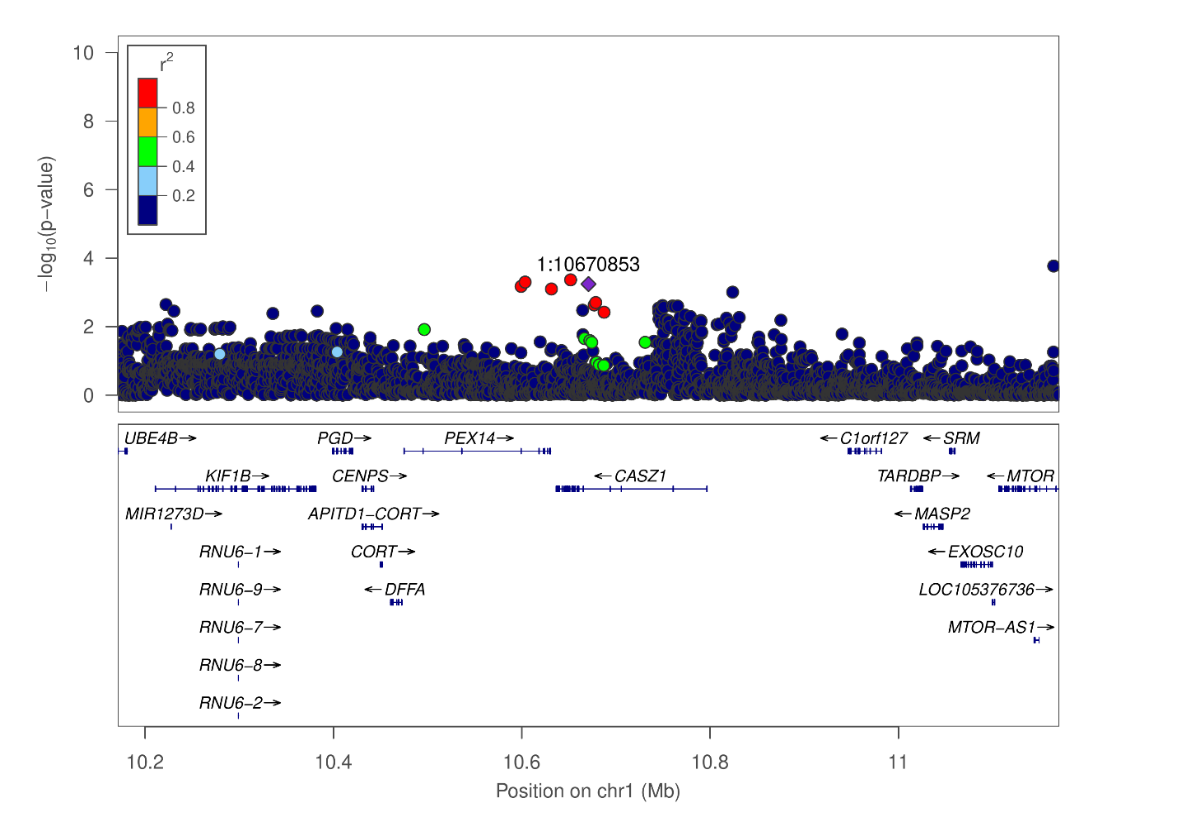


Top CHRIS SNP: rs807624 (2:15,642,347) at ***DDX1***


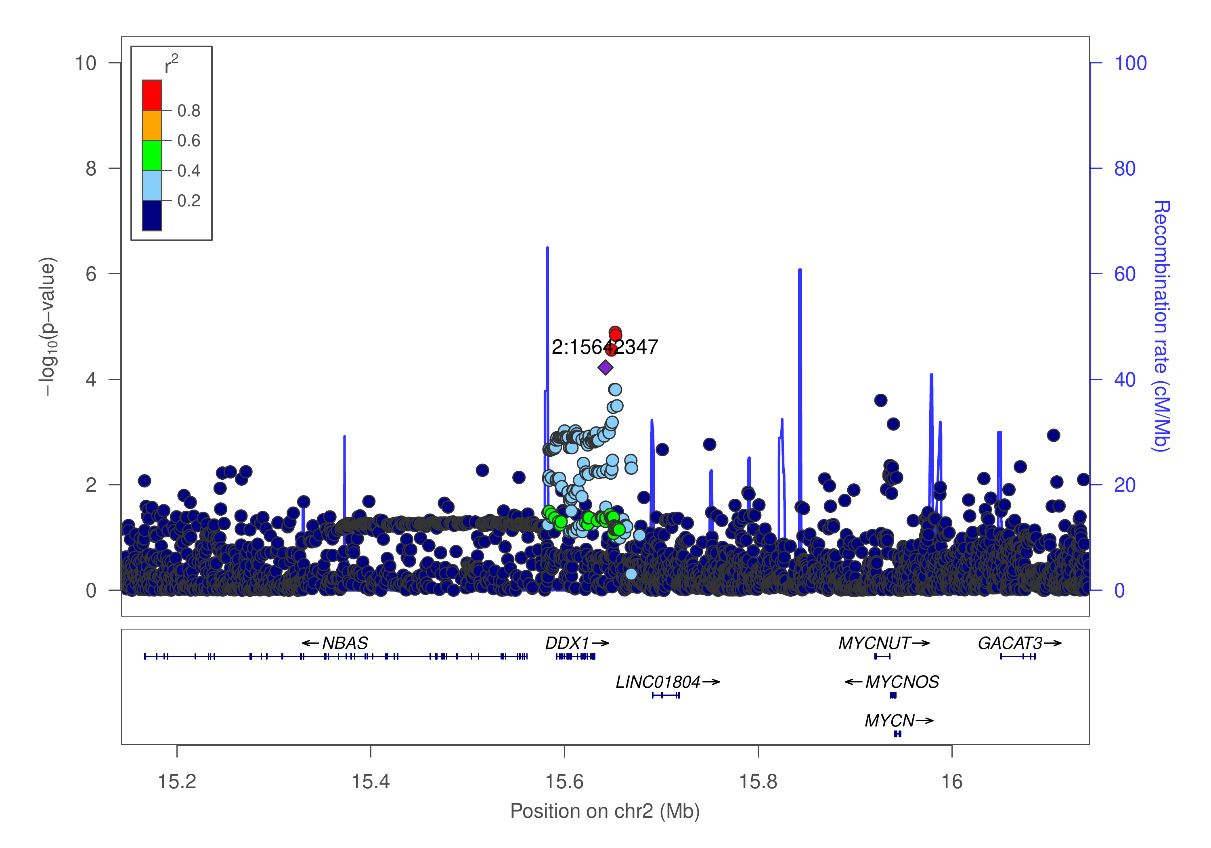


Top CHRIS SNP: rs28817415 (4:76,480,299) at ***SHROOM3***


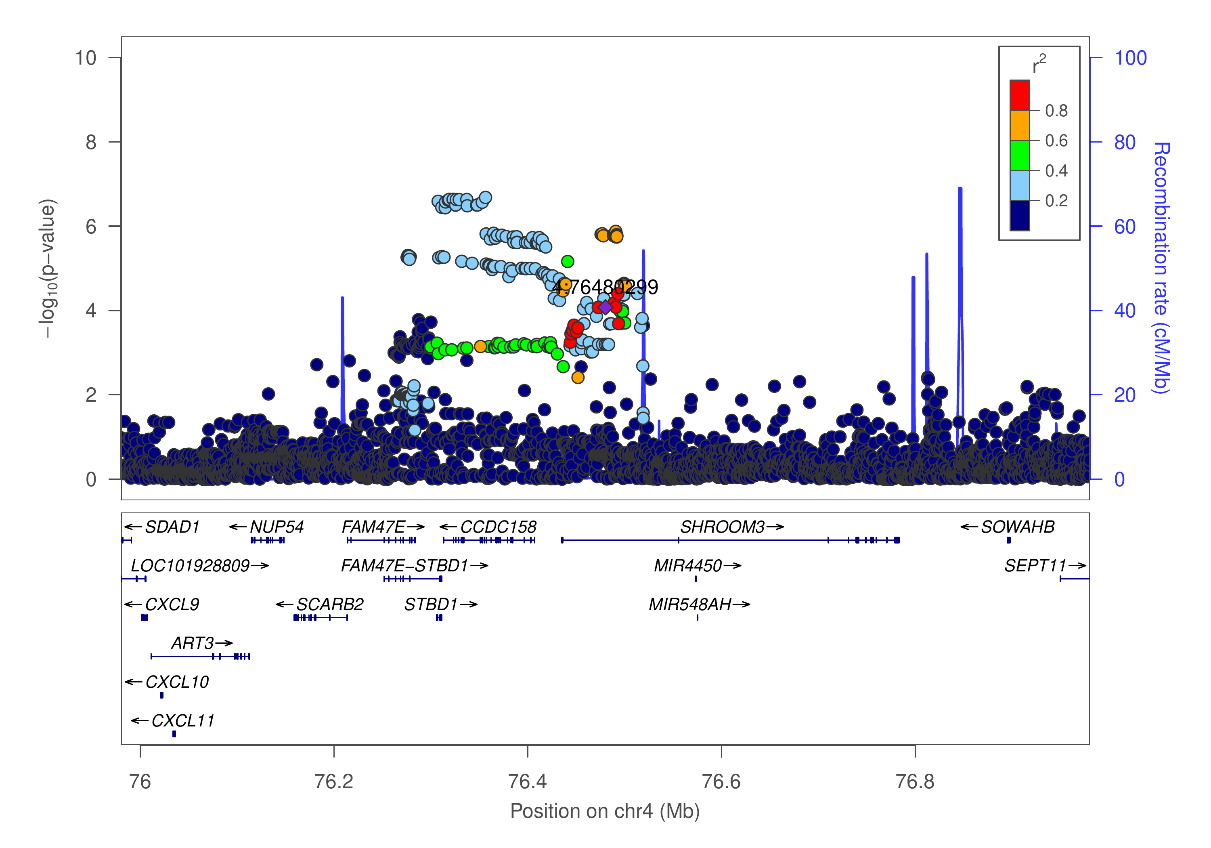


Top CHRIS SNP: rs1362800* (5:39,378,013) at ***DAB2******


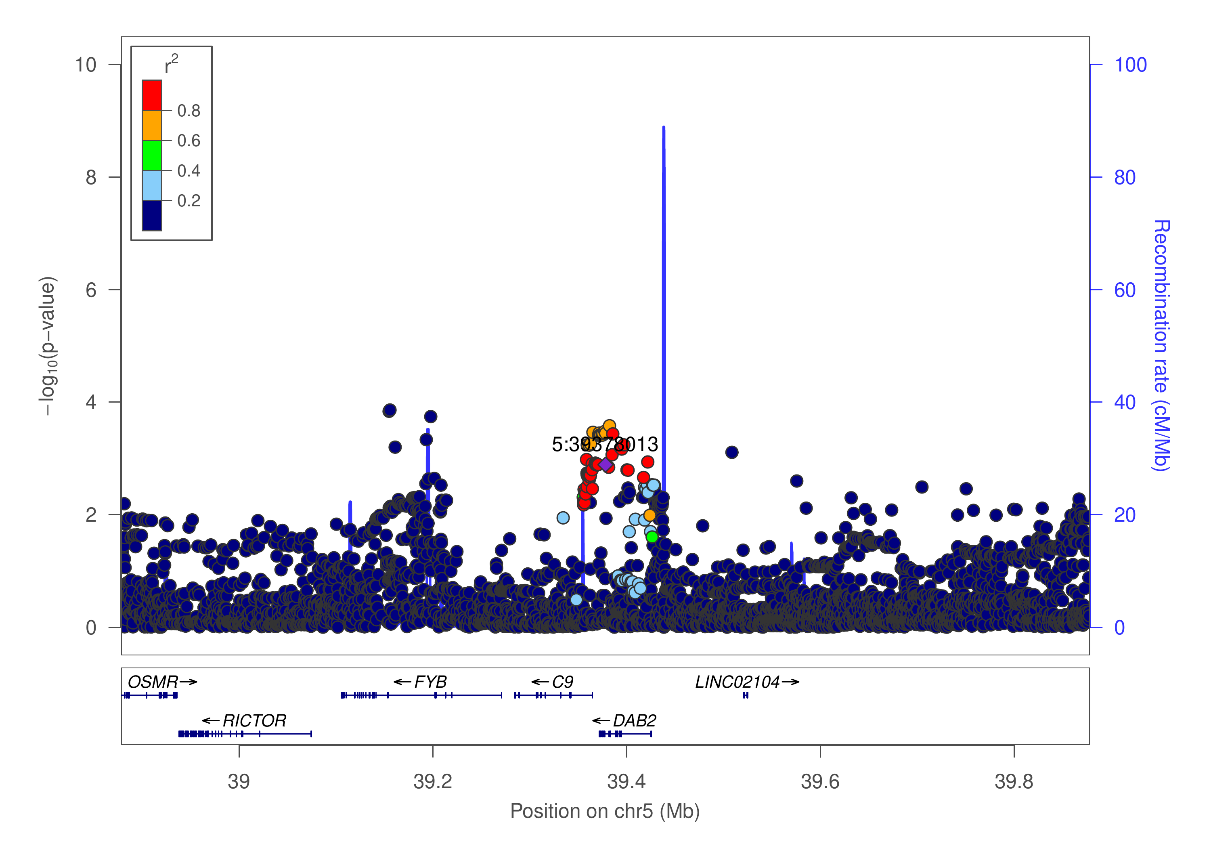


Top CHRIS SNP: rs3812036 (5:177,386,403) at ***SLC34A1***


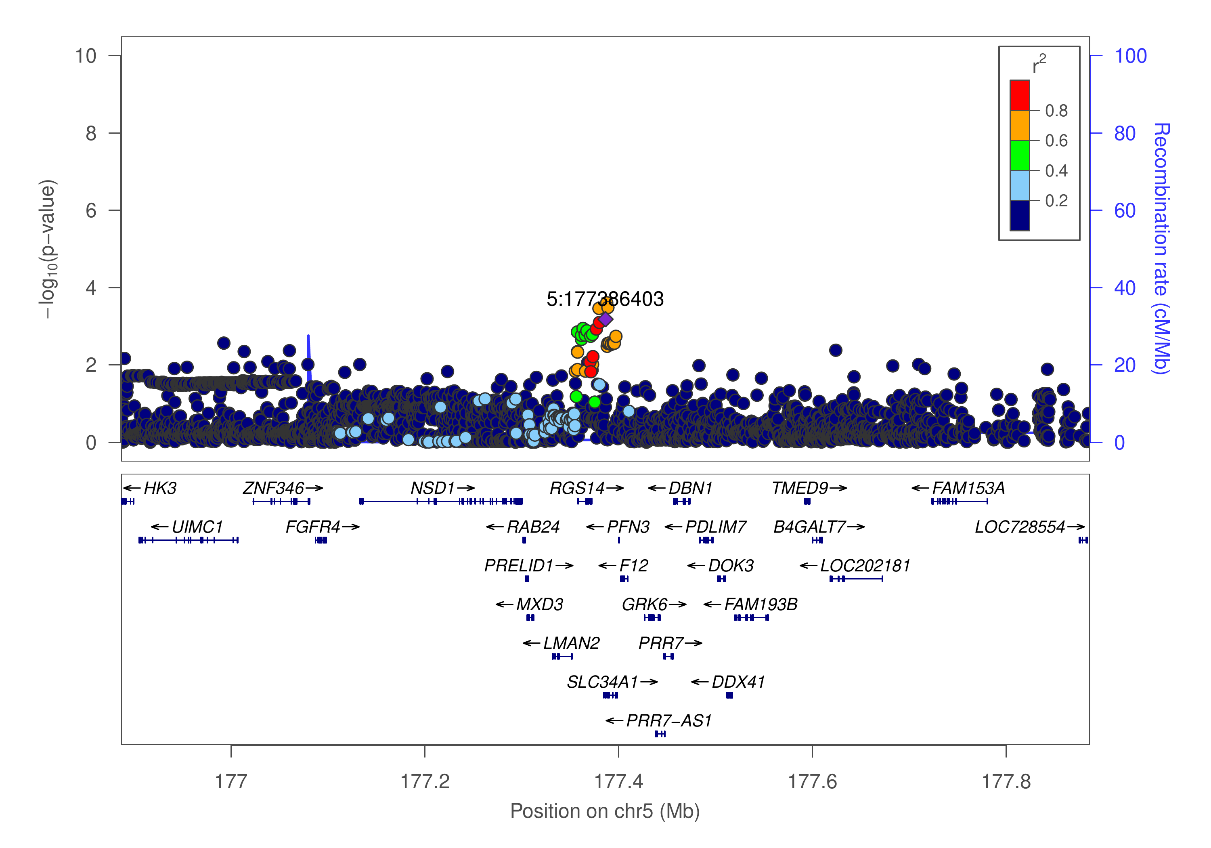


Top CHRIS SNP: rs6973656* (7:77,793,266) at ***TMEM60******


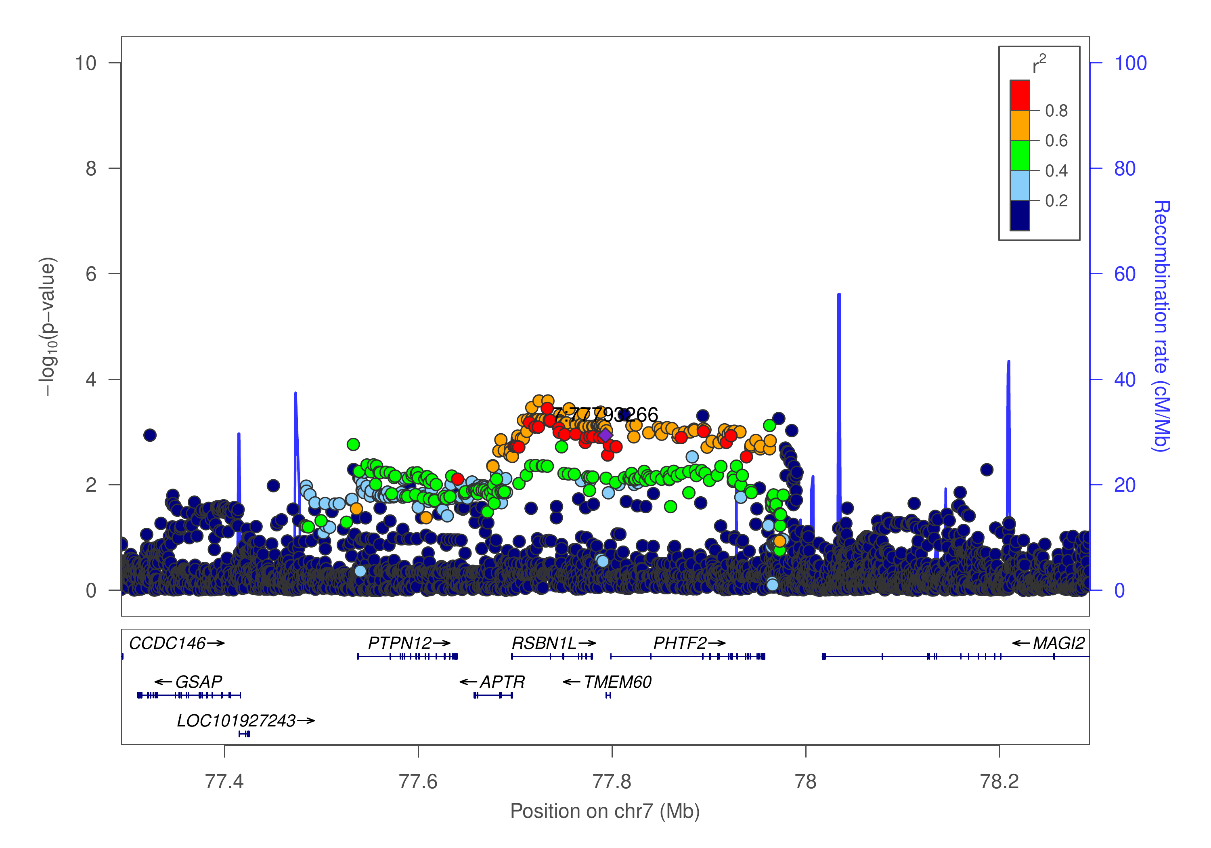


Top CHRIS SNP: rs34861762* (8:23,890,907) at ***STC1******


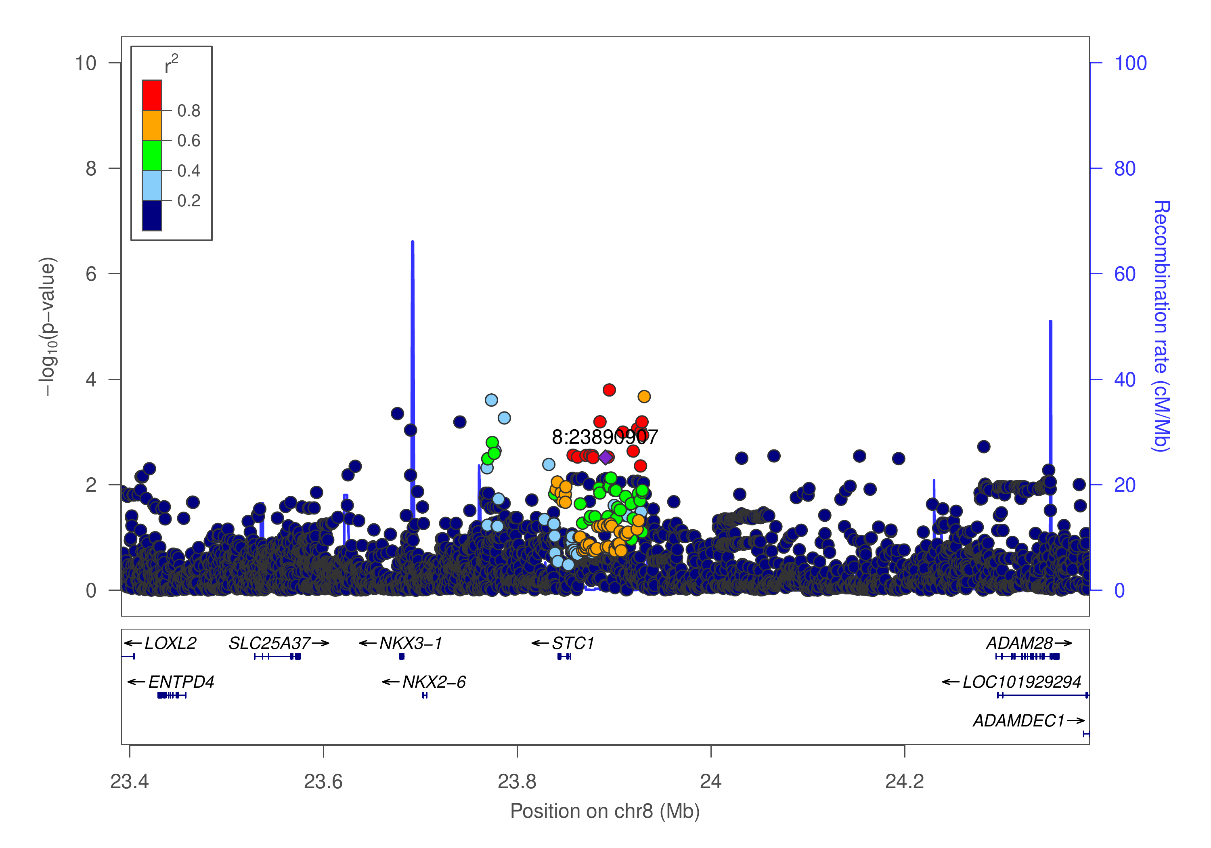


Top CHRIS SNP: rs2039424 (9:68,817,258) at ***PIP5K1B***


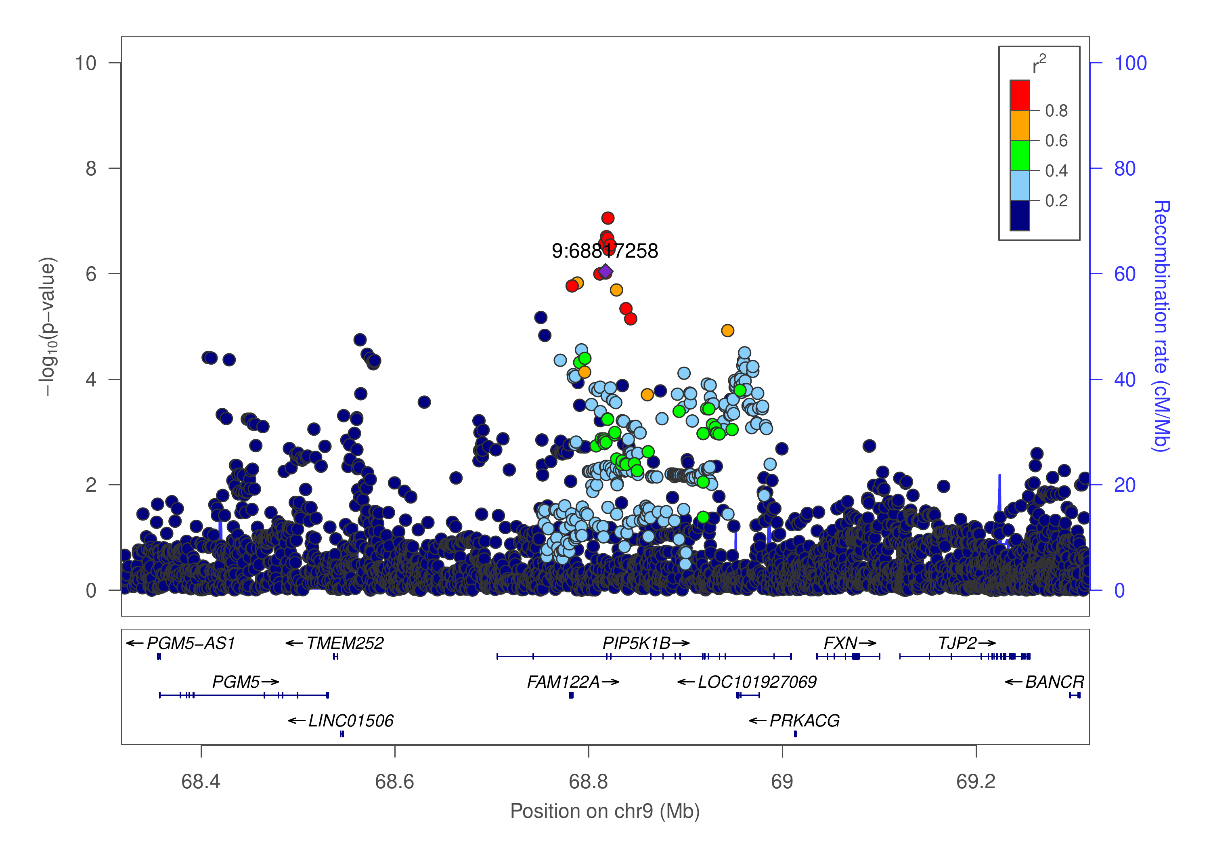


Top CHRIS SNP: rs11237450* (11:78,312,310) at ***GAB2******


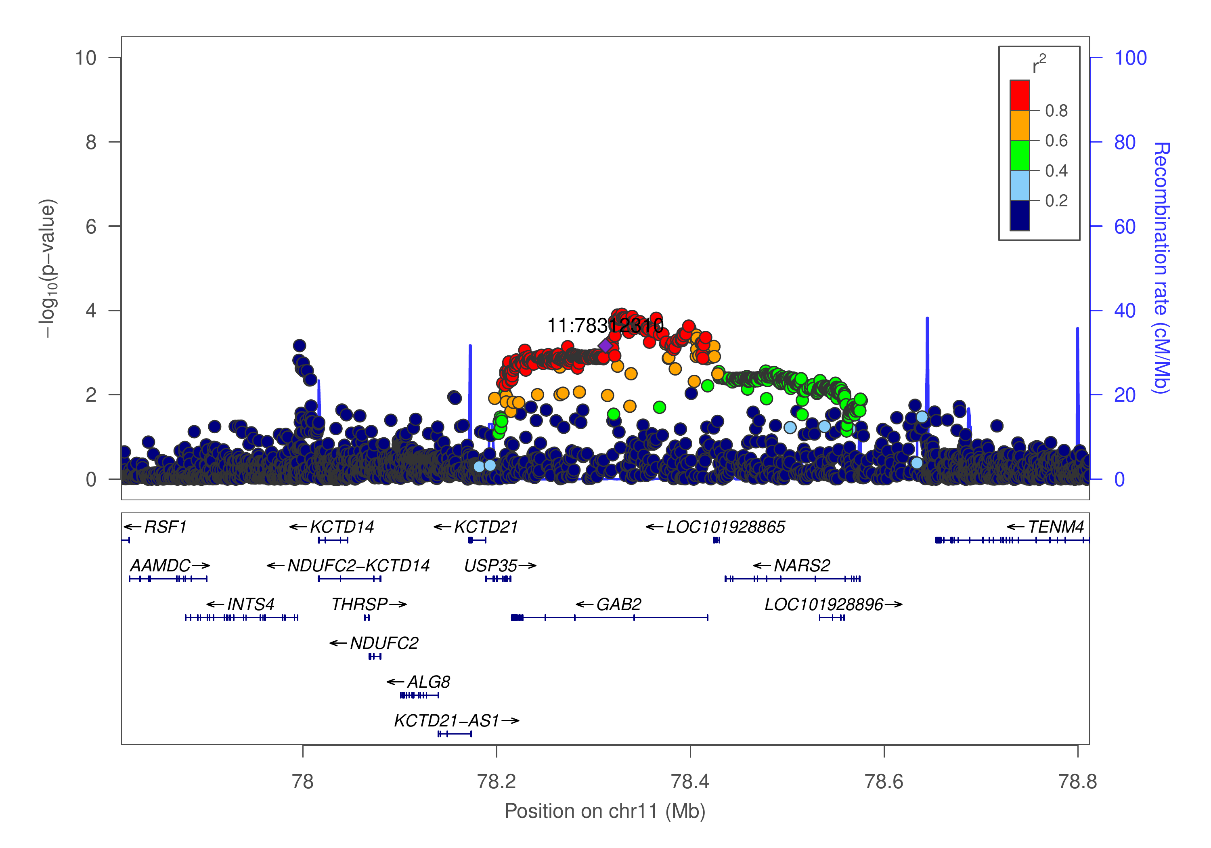


Top CHRIS SNP: rs59646751 (15:98,733,292) at ***IGF1R***
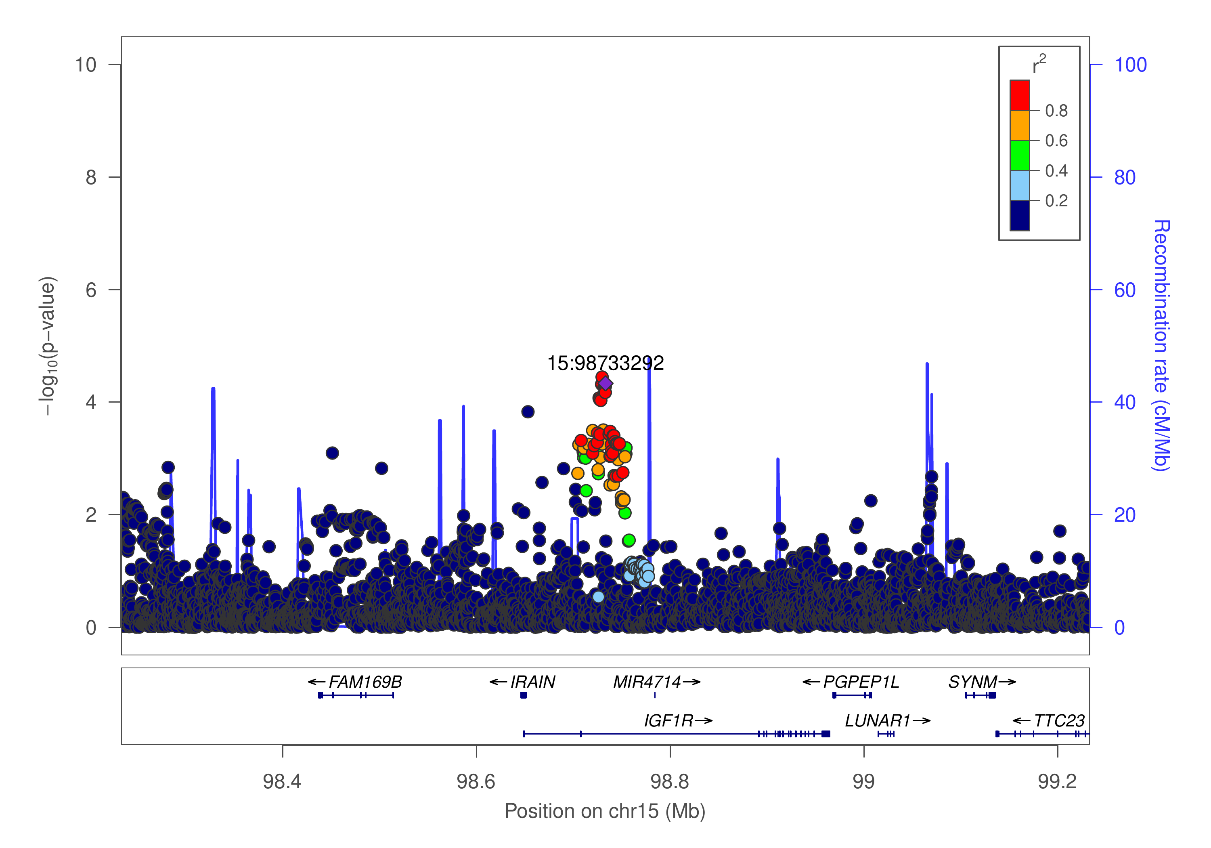


Top CHRIS SNP: rs77924615 (16:20381010) at ***UMOD-PDILT***


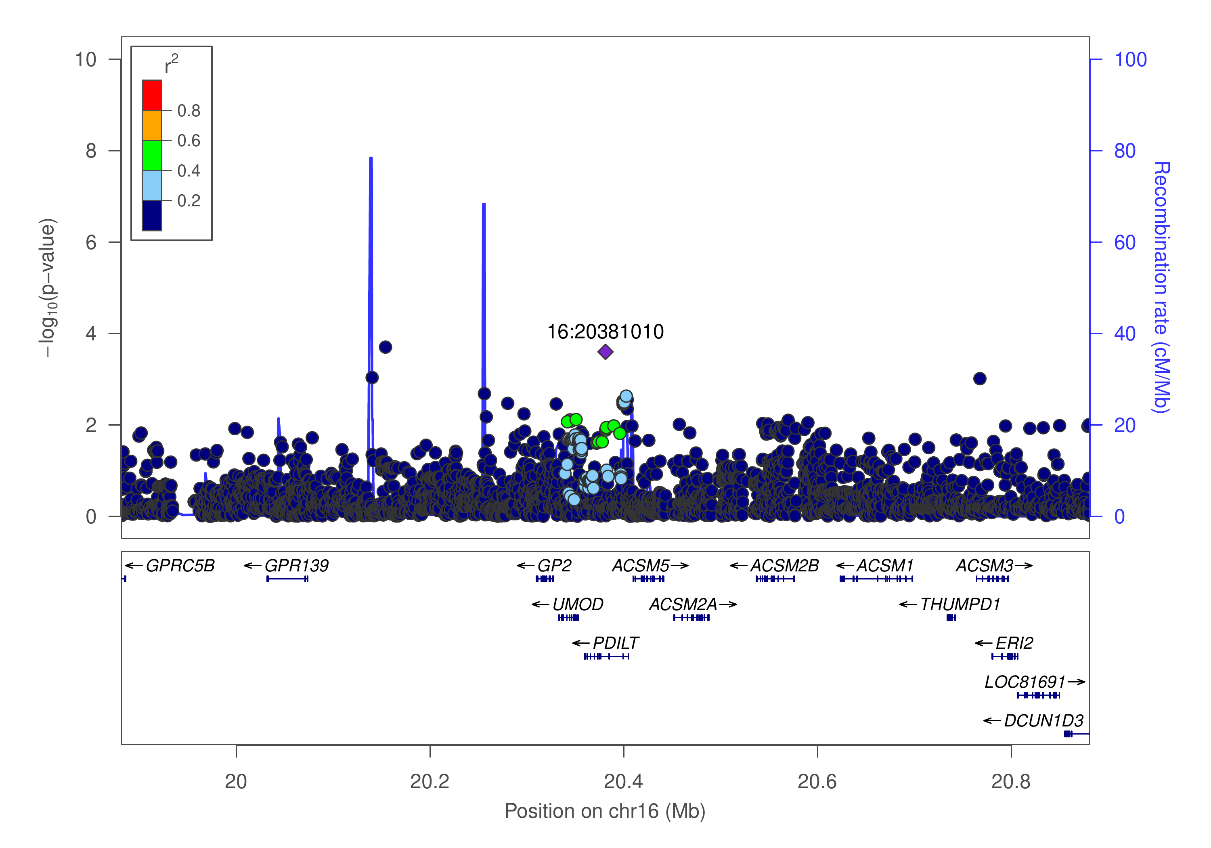


# Supplementary Figure S5. Comparison of effect size and standard error ratio for the variants in the model regressing ln(eGFRcrea) on age, sex, FT3 (or FT4), genetic variants with and without adjusting for municipality.


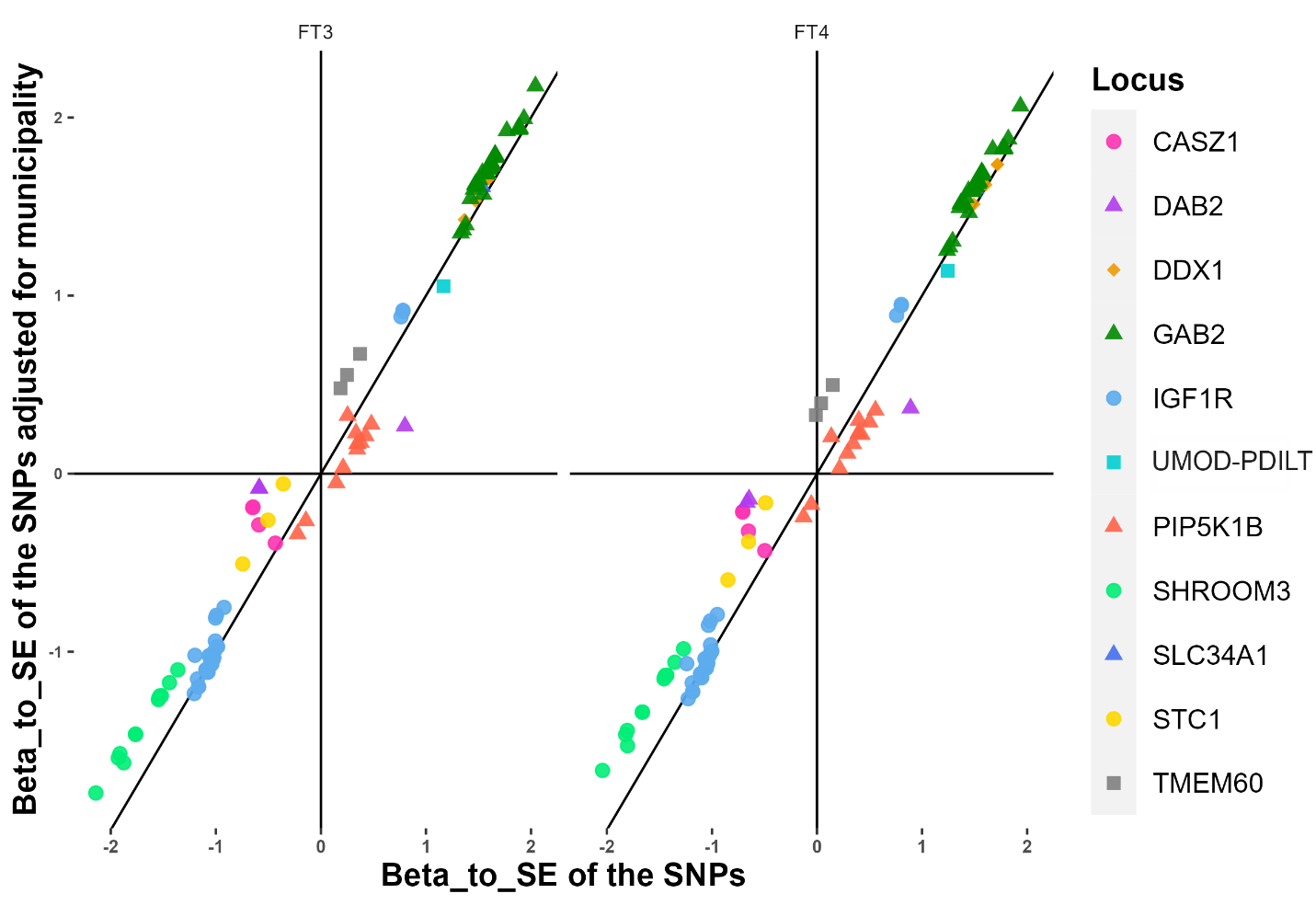


# Supplementary Figure S6. Genetic overlap between CHRIS and 1000 Genomes project samples, showing that the CHRIS study matches the European ancestry.


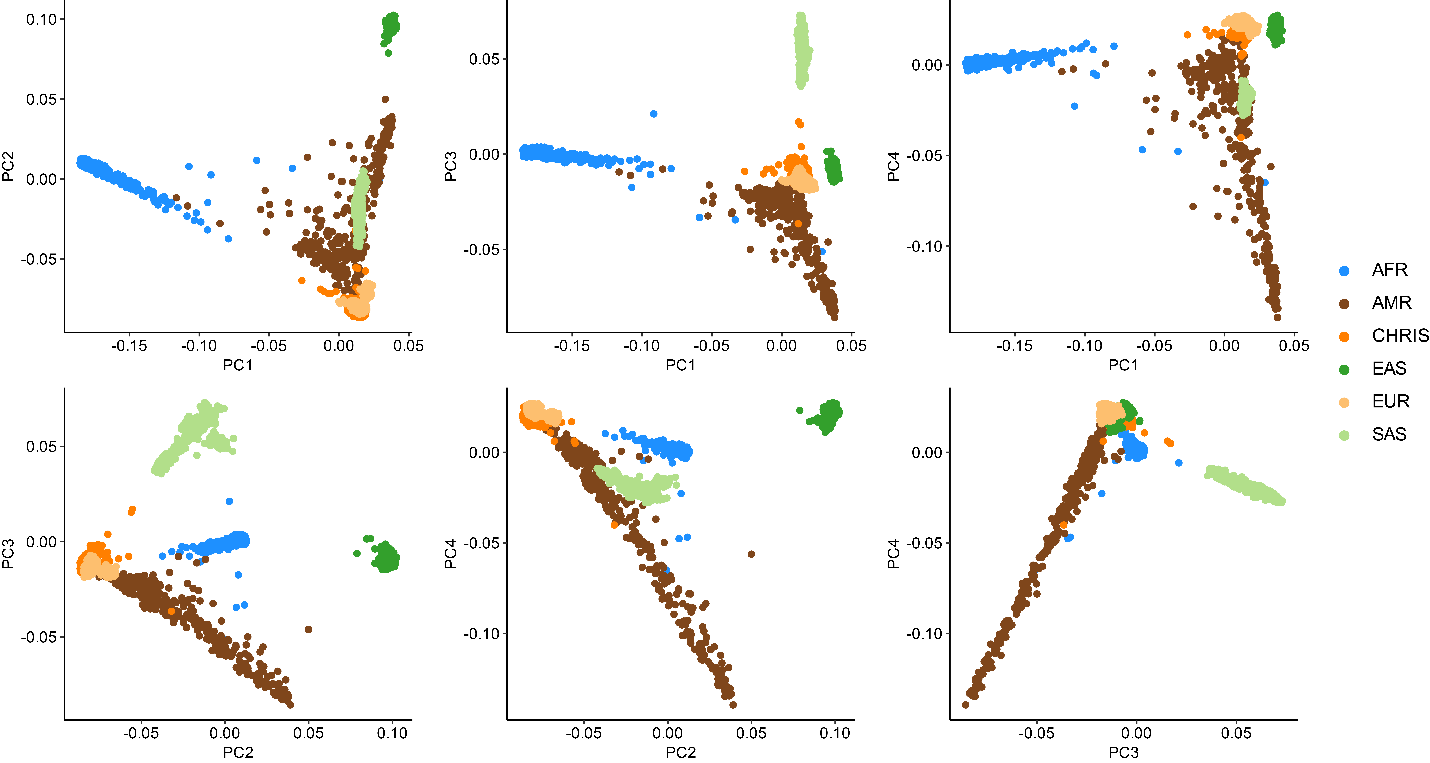

Supplement: S1 File — (DOCX) [file pone.0323057.s001.docx]
