# Supplementary material for: Systematic mediation and interaction analyses of kidney function genetic loci in a general population study
Source: PLoS One. 2025 Jun 24;20(6):e0323057. doi: 10.1371/journal.pone.0323057 (PMC12186986; doi:10.1371/journal.pone.0323057)
Supplement: Attachment — Submitted filename: rebuttal_letter.pdf [file pone.0323057.s004.pdf]

Point-by-point response to Reviewers

Reviewer #1:

This is an interesting article which takes GWAS meta analysis data and undertakes a systematic mediation to look for quantitative traits that may underlie the SNPs leading to chronic kidney disease. Some additions are needed to maximise the utility of this analysis.

> **Response**: we thank the Reviewer for their positive assessment of our work. In the following, we respond to each specific issue.

1. Page 4 - the rather convoluted text regarding replication needs to be revised / simplified. The authors say it is not a replication analysis but then refer to it as a replication analysis. I understand their meaning but it needs to be phrased more clearly.

> **Response**: We agree with the Reviewer that the use of the “replication” terminology was misleading. We re-wrote the rationale of the study (see **Introduction**, **pages 3-4, page 85-91**) which now reads as follows:
>
> > “We focused our investigation on 147 loci identified by a large GWAMA of the CKDGen Consortium with proven kidney function relevance [17]. In the cited work, the CKDGen identified 308 loci associated with eGFRcrea at genome-wide significance level; of them, 264 were replicated in an independent population study and thus validated; but given eGFRcrea might also reflect creatinine metabolism in addition to kidney function, the authors further filtered their results for direction-consistent association with blood urea nitrogen (BUN), an alternative marker of kidney function [17].”
>
> and removed the concept of replication throughout.

2. The association of variants in SLC34A1 and aPTT - this needs to be dissected further, as the authors point out the snp is near to F12 which is more likely to be biologically relevant. Some eQTL or candidate cis-regulatory elements (cCREs) analysis for the SNPs of interest should be investigated (see Loeb GB et al Nature Genetics).

> **Response**: We thank the Reviewer for their observation. We have now generated a new Table 4 including expression quantitative trait loci (eQTLs) from the GTEx Consortium version 10 and kidney tissue from the Susztak laboratory dataset, and protein QTLs (pQTLs) from the UK Biobank and DECODE genetics databases.
>
> Results illuminate further the reasons of the observed mediation. From the kidney function perspective, the most associated gene was RGS14, which plays a role in phosphate metabolism by the kidney. Thus, there are two genes under the same signal both involved in kidney phosphate handling: RGS14 and SLC34A1. On the coagulation side, we have identified an eQTL for F12 in the esophagous mucosa plus pQTLs for kininogen 1 (KNG1) and coagulation factor 2 (F2).
>
> We thus speculate that the observed statistical mediation might be the result of confounding by LD, with nearby genes acting independently on parallel pathways: one with causal effects on kidney function and one for the coagulation cascade. These observations are now proposed in an organic way through the presentation of 6 possible

causal scenarios, presented in the **new Figure 5**. Among the 6 possible scenarios we also discuss potential collider bias (**in response to Reviewer 2, point 4**).

Please, find **new Table 4, new Figure 5, new Supplementary Tables 8, 9, 10, and 11**, and new text in the **Methods**, where we introduce the eQTL and pQTL lookups (**page 7, lines 210-217**):

> "We queried variants for which we observed potentially mediated effects in the European ancestry datasets of the GTEx Consortium v10 database (https://gtexportal.org/home/; 1-Mar-2025) across 49 tissues (n=65 to 573 samples per tissue) using GTEx API v2 and in the Human Kidney eQTL data [34] (n=686), to assess association with gene expression (eQTL) at P<5×10-8. To identify protein quantitative trait loci (pQTLs) at P<5×10-8, we interrogated GWAS summary results for 2923 plasma proteins on 54,219 UK Biobank participants [35] and 4502 whole blood proteins from a deCODE genetics study [36] (n=35,559; https://decode.com/summarydata; 1-Mar-2025)."

In the **Results**, where we discuss findings from the eQTL and pQTL investigations (**page 10, lines 308-312**):

> "At SLC34A1, the partial mediation of aPTT corresponded to a 21% larger effect of the SNP on ln(eGFRcrea). eQTL investigation highlighted association with RGS14 expression across most tissues (Table 4). The involved genetic variant was an eQTL for coagulation factor 12 (F12) in the esophagous mucosa and a pQTL for kininostatin, a fragment of kininogen 1 (KNG1), and coagulation factor 2 (F2), all involved in the coagulation cascade (Table 4)."
>
> and in the **Discussion**, **page 12, lines 367-372**:
>
> "Statistical mediation observed at SHROOM3 and SLC34A1 is compatible with different causal scenarios (Fig 5). First, given several established connections between both loci and eGFRcrea, it looks implausible that genes and eGFRcrea may independently affect the mediators, generating apparent mediation (Fig 5, scenario 1). Moreover, given similar allele frequencies between CHRIS and CKDGen, collider bias acting through population selection seems implausible (Fig 2A and 2D)."
>
> and **pages 13-14, lines 398-412**:
>
> "At SLC34A1, confounding by LD seems the most likely possibility. Both RGS14 [52] and SLC34A1 [53] are involved in phosphate handling that is critically dealt by the kidney, also in response to parathyroid hormone (PTH) regulation, as confirmed by our pQTL lookup that identified association with PTH in the deCODE proteomics data. The association with eGFRcrea [38] likely reflects causal involvement of this locus in kidney function regulation. In addition, the locus was previously associated with aPTT in 9240 European ancestry individuals [39] including participants from the same area where the CHRIS study was sampled. By quantifying the clotting time from the activation of factor XII (F12) until the formation of a fibrin clot, aPTT is a measure of the integrity of the intrinsic and common coagulation pathways [54]. The observed mediation, in which adjustment for aPTT increased the SNP-eGFRcrea association by 21%, is likely related to the proximity between SLC34A1 and the coagulation factor gene F12. F12 initiates the coagulation cascade, which in turn involves kininogen 1 (KNG1) and coagulation

factor 2 (F2), both of which were identified in our pQTL lookup. Results are thus compatible with the presence of common haplotypes partially tagging both RGS14/SLC34A1 and F12, inducing partially overlapping effects on eGFRcrea and aPTT."

3. The PDILT snp is usually associated with UMOD - can this be mentioned clarified

   Response: We replaced PDILT with UMOD-PDILT throughout.

4. The SHROOM3 findings related to Magnesium are interesting and perhaps a mechanistic diagram would help here to understand how SHROOM3 might be mediating magnesium regulation. Again for the SHROOM3 snp some QTL or candidate cis-regulatory elements (cCREs) analysis for the SNPs of interest should be investigated.

   Response: To respond to this point, we can largely **refer to the response to your point #2 above**. For the specific results pertaining to the *SHROOM3* locus, we now provide eQTL and pQTL evidence in the **new Table 4**, and discuss the causal scenarios that might have originated the evidence of mediation in the **new Figure 5** and related discussion.

   In addition to what already reported in previous point 2, which was common to both SHROOM3 and SLC34A1 loci, please, find new text related to SHROOM3 in the **Results (page 10, lines 301-307)**:

   > "At SHROOM3, the partial mediation of Mg corresponded to about 11% effect attenuation (Table 3). eQTL investigation across all genes located in the SHROOM3 locus did not identify any genome-wide significant association with SHROOM3 expression (Table 4), as it would be expected given the developmental nature of the gene [41, 42]. However, SHROOM3 resulted being the most associated gene in the kidney tissue. The variants involved in the mediation were also associated with cystatin C protein levels and with other kidney-function relevant proteins such as NAR3 and CXCL11 (I-TAC) [43] (Table 4)."

   And in the **Discussion**, **pages 12-13, lines 372-397**:

   > "At SHROOM3, apparent mediation due to LD confounding is possible: there are several haplotypes spanning all genes in the locus that were jointly associated with eGFRcrea and Mg [43]. However, it is unclear which of such genes might affect Mg without jointly affecting eGFRcrea. Known biology should also bring to exclude a causal effect of Mg on eGFRcrea. For SHROOM3, the most likely scenario looks like that of SHROOM3 having independent causal effects on eGFRcrea and Mg levels. Genetic variants at SHROOM3 have been associated with CKD [8, 9], reduced eGFRcrea [9], increased albumin-to-creatinine ratio [10], and low serum magnesium levels [11]. SHROOM3 is necessary to maintain the glomerular filtration barrier integrity [41]. Variants in this gene have been shown to be associated with increased risk of CKD through disruption of the transcription factor TCF7L2 in podocyte cells [48]. The nearby CCDC158 is involved in renal proximal tubular endocytosis and is expressed in renal proximal tubular cells and in glomeruli of individuals free from nephrocalcinosis [49]. There is thus support for a causal role of the SHROOM3 locus on kidney function, at least through SHROOM3 itself, but not excluding CCDC158. On the magnesium side, one can note that SHROOM3 is also expressed in the epithelia morphogenesis of the gut [50], which

has a crucial role in Mg absorption and regulation. This evidence would be compatible with a previous observation that Mg association with the SHROOM3 locus did not change when adjusting for eGFRcrea, suggesting pleiotropic independent effects [40]. However, being serum magnesium a poor marker of dietary magnesium [51] this hypothesis should be considered with caution. On the other hand, our observation of a partial mediation (Mg adjustment caused 11% reduction of SHROOM3 effect on eGFRcrea), would be compatible with the kidney's role on Mg regulation, through excretion and reabsorption. By this reasoning, high Mg levels could be partially determined by long-term lower kidney function levels due to SHROOM3 variations affecting embryonal kidney development. Altogether, combining Fig 5's possible scenario 3 and likely scenario 6 would imply the partial mediation as the result from both a direct effect of SHROOM3 on Mg and an indirect effect of SHROOM3 on Mg through lower kidney function."

5. It would be useful to know if any of the SNPs are in coding regions.

Response: We added VEP annotation of all 163 variants to Suppl. Tab 3.

6. The associations with abnormal thyroid function as modifiers are really interesting and that adds to the novelty of this study

Response: We thank the Reviewer for this comment. It was also our impression and having their confirmation is important.

Reviewer #2:

Ghasemi-Semeskandeh et al., in this study of the CHRIS cohort (n=10,146), replicated associations for 11 kidney function loci, showing effect sizes up to 5.4 times larger than those observed in the CKDGen kidney function meta-GWAS involving 1 million individuals. Mediation analysis highlighted serum magnesium and activated partial thromboplastin time as partial mediators at SHROOM3 and SLC34A1, respectively. Additionally, thyroid function was identified as a genetic effect modifier, with significant SNP-by-TSH interactions at STC1. These findings underscore the value of individual population studies in elucidating genetic mechanisms underlying kidney function. However, I have several suggestions for the authors to consider:

Response: We thank the Reviewer for their positive assessment of our work. We provide point-by-point response to each issue below.

1.Ethical Statement Placement: In the Methods section, I suggest beginning with the Ethical Statement, which is currently placed at the end of the section, for improved organization and clarity.

Response: To improve clarity, we moved the Ethical statement to the beginning of the **Methods**, next to the study description (**page 4, lines 110-112**).

2. Selection of Loci: The authors selected 147 kidney function loci from over 264 independent loci reported in the CKDGen study. Please clarify how this number was reduced to 147 after

excluding CHRIS samples from the GWAS summary statistics. Additionally, it is unclear how MetaSubtract was used to exclude CHRIS data from the summary statistics. Why not conduct the analysis solely on independent CHRIS subjects (n=~10,000), even if the sample size is smaller, to ensure cleaner results?

Response: We thank the Reviewer for highlighting this point, which was unclear. The CKDGen validated 264 loci in association with eGFRcrea, but only 147 of them demonstrated a direction consistent association with blood urea nitrogen, ie an alternative marker of kidney function. This aspect is relevant because, given the CHRIS study lacks kidney function markers other than eGFRcrea, there wouldn't have been another way to ensure the kidney function relevance of the study results. We have expanded the rationale of locus selection in the **Introduction**, **page 3-4, lines 85-91**, which reads as follows:

"We focused our investigation on 147 loci identified by a large GWAMA of the CKDGen Consortium with proven kidney function relevance [17]. In the cited work, the CKDGen identified 308 loci associated with eGFRcrea at genome-wide significance level; of them, 264 were replicated in an independent population study and thus validated; but given eGFRcrea might also reflect creatinine metabolism in addition to kidney function, the authors further filtered their results for direction-consistent association with blood urea nitrogen (BUN), an alternative marker of kidney function [17]."

Concerning the second part of the question, we can clarify that we did analyze all available 10,146 CHRIS samples, including those that were previously included in CKDGen. But, in order to compare CHRIS results against CKDGen results, we needed first to clean CKDGen results from the CHRIS effects, to ensure independency of the two samples being compared. This can be conveniently done using MetaSubtract by providing: (1) the GWAS meta-analysis results (CKDGen, in this case) and (2) the GWAS results of the contributing study (the CHRIS study, in this case). Given a meta-analysis is, in essence, a weighted average, knowing one of the addends is enough to formally remove it from the aggregated meta-analysis results. Please, find more details in the MetaSubtract reference paper (Nolte IM. Metasubtract: an R-package to analytically produce leave-one-out meta-analysis GWAS summary statistics. Bioinformatics 2020; 36:4521-2, doi:10.1093/bioinformatics/btaa570). In the **Methods**, **page 6, lines 161**, we added

"to ensure sample independence"

to a reinforce the motivation of excluding CHRIS effects from CKDGen.

An alternative way could have been to conduct a GWAS of eGFRcrea in the CHRIS study. Given the limited sample size, the study might have been underpowered to identify relevant loci (for instance, notice that the P-value for association at the UMOD-PDILT locus was 2.53×10^{-4} in CHRIS). In addition, the study would have needed to seek for external replication (again, the CKDGen results after removing previously used CHRIS samples) and, having only eGFRcrea as a marker of kidney function, validation of kidney function relevance through comparison with an alternative marker (again from an external dataset). Altogther, this looked to us as a much more complex procedure than starting from loci that were already solidly identified and validated with regard to kidney function relevance.

3. Correction for Multiple Testing: Did the authors account for multiple testing in the mediation analysis, given that 70 quantitative traits were assessed? While multiple testing correction for

SNPs was mentioned, clarifying this point for the quantitative trait analysis would strengthen the study.

> **Response**: Yes, we did. Please, see **Methods**, **page 7, lines 197-199**, which read as follows:
>
> “the SNP is also associated with the trait (step 3) at a multiple-testing corrected level of 6.5×10^{-5} (corresponding to 0.05 / (70 traits × 11 independent loci))”

4. Evaluation of Collider Bias: Did the authors assess the potential for collider bias during mediation analysis, particularly for the quantitative traits? Addressing this would help validate the findings further.

> **Response**: We thank the Reviewer for raising this important point. We recognize that the observed evidence of statistical mediation might have result from some types of epidemiological bias rather than true biological mechanisms. To discuss the results more objectively, we have now introduced a **new Figure 5**, which displays and discuss 6 potential scenarios, including collider bias and confounding by linkage disequilibrium, in addition to alternative causal mechanisms.
>
> The Discussion on the causal mechanisms has been illuminated by additional exploration of eQTLs and pQTLs that we conducted in response to **Reviewer 1, points 2 and 4**. Please, see those points for completeness. Most importantly, please see the **new text** in the **Discussion**, **pages 12-14, lines 367-412**:
>
> > “Statistical mediation observed at SHROOM3 and SLC34A1 is compatible with different causal scenarios (Fig 5). First, given several established connections between both loci and eGFRcrea, it looks implausible that genes and eGFRcrea may independently affect the mediators, generating apparent mediation (Fig 5, scenario 1). Moreover, given similar allele frequencies between CHRIS and CKDGen, collider bias acting through population selection seems implausible (Fig 2A and 2D). At SHROOM3, apparent mediation due to LD confounding is possible: there are several haplotypes spanning all genes in the locus that were jointly associated with eGFRcrea and Mg [43]. However, it is unclear which of such genes might affect Mg without jointly affecting eGFRcrea. Known biology should also bring to exclude a causal effect of Mg on eGFRcrea. For SHROOM3, the most likely scenario looks like that of SHROOM3 having independent causal effects on eGFRcrea and Mg levels. Genetic variants at SHROOM3 have been associated with CKD [8, 9], reduced eGFRcrea [9], increased albumin-to-creatinine ratio [10], and low serum magnesium levels [11]. SHROOM3 is necessary to maintain the glomerular filtration barrier integrity [41]. Variants in this gene have been shown to be associated with increased risk of CKD through disruption of the transcription factor TCF7L2 in podocyte cells [48]. The nearby CCDC158 is involved in renal proximal tubular endocytosis and is expressed in renal proximal tubular cells and in glomeruli of individuals free from nephrocalcinosis [49]. There is thus support for a causal role of the SHROOM3 locus on kidney function, at least through SHROOM3 itself, but not excluding CCDC158. On the magnesium side, one can note that SHROOM3 is also expressed in the epithelia morphogenesis of the gut [50], which has a crucial role in Mg absorption and regulation. This evidence would be compatible with a previous observation that Mg association with the SHROOM3 locus did not change when adjusting for eGFRcrea, suggesting pleiotropic

independent effects [40]. However, being serum magnesium a poor marker of dietary magnesium [51] this hypothesis should be considered with caution. On the other hand, our observation of a partial mediation (Mg adjustment caused 11% reduction of SHROOM3 effect on eGFRcrea), would be compatible with the kidney's role on Mg regulation, through excretion and reabsorption. By this reasoning, high Mg levels could be partially determined by long-term lower kidney function levels due to SHROOM3 variations affecting embryonal kidney development. Altogether, combining Fig 5's possible scenario 3 and likely scenario 6 would imply the partial mediation as the result from both a direct effect of SHROOM3 on Mg and an indirect effect of SHROOM3 on Mg through lower kidney function.

At SLC34A1, confounding by LD seems the most likely possibility. Both RGS14 [52] and SLC34A1 [53] are involved in phosphate handling that is critically dealt by the kidney, also in response to parathyroid hormone (PTH) regulation, as confirmed by our pQTL lookup that identified association with PTH in the deCODE proteomics data. The association with eGFRcrea [38] likely reflects causal involvement of this locus in kidney function regulation. In addition, the locus was previously associated with aPTT in 9240 European ancestry individuals [39] including participants from the same area where the CHRIS study was sampled. By quantifying the clotting time from the activation of factor XII (F12) until the formation of a fibrin clot, aPTT is a measure of the integrity of the intrinsic and common coagulation pathways [54]. The observed mediation, in which adjustment for aPTT increased the SNP-eGFRcrea association by 21%, is likely related to the proximity between SLC34A1 and the coagulation factor gene F12. F12 initiates the coagulation cascade, which in turn involves kininogen 1 (KNG1) and coagulation factor 2 (F2), both of which were identified in our pQTL lookup. Results are thus compatible with the presence of common haplotypes partially tagging both RGS14/SLC34A1 and F12, inducing partially overlapping effects on eGFRcrea and aPTT."

5. Discrepancy in Variant Count: The authors mention testing 147 variants, but in the Results section (Page 9, Line 256), they state that 163 variants were tested at 11 loci. Are these independent SNPs? Please clarify this discrepancy and provide a detailed explanation.

Response: CKDGen reported 147 loci associated with kidney function and CKD in the European ancestry. 11 loci among the 147 resulting in significant association in CHRIS. For each of these 11 loci, we retained proxy variants in strong LD (r^2>0.8) with the sentinel variant. In total, we found 163 variants in-LD with sentinel variants across 11 loci. This has now been clarified better in the **Results, page 9, lines 250-252**:

"For each such locus we retained all variants in LD (r2>0.8) with the lead SNP, totaling 163 mostly intronic or non-coding exonic variants over the 11 loci (S3 Table in File S2)."

6. Figure Legends: Some figures lack clear explanations in their legends, making it difficult to understand what tests were conducted. Providing detailed figure legends will significantly improve comprehension.

Response: We edited and expanded all figure legends to improve clarity. We hope they can now deliver the significance of the results in a better way. Please, note the following changes:

- **Figure 2** has been modified to incorporate all genetic comparison between the CHRIS study and European-ancestry results. The figure now includes 4 panels: panel A shows a new principal component plot showing that the CHRIS study fully overlap the 1000 Genomes EUR population (in response to **your point 7, below**); panel B corresponds to previous Figure 2; panels C and D incorporate previous Figure 3 A and B.
- **Figure 3** corresponds to previous Figure 4
- **Figure 4** corresponds to previous Figure 5, where we improved the legend and explanation
- We provide a **new Figure 5** describing the potential causal models, including possible collider bias and confounding, that could have produced evidence of statistical mediation (in response to **your point no. 4**).

7. PCA Plot for Ancestry: To enhance clarity, consider including a PCA plot of the CHRIS cohort alongside the 1000 Genomes dataset to better illustrate ancestry composition.

Response: We thank the Reviewer for proposing this analysis that enhances clarity and helps contextualize the entire analysis within the European-ancestry context.

We conducted a PCA of CHRIS and the 1000 Genomes project data, as described in the **Methods** (**page 5, lines 141-151**):

"Genetic principal component analysis (PCA)

We performed PCA on autosomal variants from the 1000 Genomes Project Phase 3 (1000GPh3) and the CHRIS study to assess population structure. 1000GPh3 VCF files for chromosomes 1 to 22 were downloaded, and a list of target variant sites was extracted from the CHRIS study genotype dataset. Variants from the 1000GPh3 dataset matching these sites were extracted using vcftools, and chromosome-specific VCF files were merged into a single dataset. To obtain a set of independent SNPs, linkage disequilibrium (LD) pruning was conducted using PLINK (--indep-pairwise 100kb 5 0.1), retaining variants with $r^2<0.1$ within 100-kb windows. PCA was conducted on the pruned dataset, extracting per-SNP weights for the first 20 PCs. Individuals from both datasets were projected onto the PC space using PLINK's --score function, generating scores for each individual for the first four PCs."

And commented in the **Results (page 9, lines 252-254)**:

"All 11 loci exhibited very similar LD pattern in CHRIS as in CKDGen (S4 Fig in S1), consistent with shared European-ancestry background (Fig 2A)."

Results are now displayed in **new Figure 2A**.

These analyses were conducted by our colleague Dr. Mohamed S. Sarhan, who has now been added as a new coauthor of this manuscript.

8. Overall, addressing these points will enhance the clarity, organization, and rigor of the study.

Response: We agree and thank the Reviewer for their constructive comment.

Reviewer #3:

The manuscript titled "Systematic mediation and interaction analyses of kidney function genetic loci in a general population study" explores the genetic factors influencing kidney function, specifically focusing on chronic kidney disease. The authors conducted a study using data from the Cooperative Health Research In South Tyrol (CHRIS) involving 10,146 participants to assess the association of 147 kidney function-related genetic loci with estimated glomerular filtration rate (eGFR) based on serum creatinine levels. The manuscript addresses a topic of great clinical importance, the relationship between genetic factors and kidney function, especially in a specific population context. The identification of genetic loci associated with glomerular filtration rate (GFR) may contribute to understanding the genetic basis of chronic kidney disease (CKD) and its implications for treatment and prevention.

Response: We thank the Reviewer for their valuable feedback and provide response to each point below.

1. Introduction

The introduction could be more robust in contextualizing the problem. It would be useful to include a literature review explaining the relevance of the genetic loci chosen and how they relate to kidney function and chronic kidney disease.

Response: We thank the Reviewer for this recommendation, but clarify that we did not make any *a priori* selection of any subgroup of loci. In fact, we considered all 147 genomic loci that the CKDGen Consortium (Wuttke 2019) had previously validated as relevant to kidney function. Their selection was very stringent: out of 308 loci resulting genome-wide significantly associated with eGFRcrea, they replicated 264 loci by means of further testing in an independent population study; of those 264, 147 resulted associated to two kidney function markers, namely eGFRcrea and blood urea nitrogen, in a direction consistent manner. Given eGFRcrea does reflect both kidney function and muscle mass metabolism, given no other kidney function marker was available in the CHRIS study, instead of working with all 264 replicated loci, we chose to focus on the subset of 147 loci that were already validated as kidney function relevant. We revised this rationale in the **Introduction** (**page 3-4, lines 85-91**):

> “We focused our investigation on 147 loci identified by a large GWAMA of the CKDGen Consortium with proven kidney function relevance [17]. In the cited work, the CKDGen identified 308 loci associated with eGFRcrea at genome-wide significance level; of them, 264 were replicated in an independent population study and thus validated; but given eGFRcrea might also reflect creatinine metabolism in addition to kidney function, the authors further filtered their results for direction-consistent association with blood urea nitrogen (BUN), an alternative marker of kidney function [17].”

It is also worth noting that most of the 11 loci showing significant effects in the CHRIS study were known since long time (e.g.: UMOD-PDILT, SHROOM3, GAB2) and providing a review here seems a bit out of scope to us. However, in response to Reviewer 1, we have now expanded discussion on functional mechanisms behind SHROOM3 and

SLC34A1, with regard to the mediation effects observed for serum magnesium and aPTT. Please, see our **response to Reviewer 1, points 2 and 4**.

2. Statistical Issues:

   The statistical analysis is adequate, but the choice to penalize the significance level for multiple tests must be clearly justified.

   Response: We penalized the significance level for multiple testing in two occasions:

   (1) at the beginning, when we assessed how many of the 147 CKDGen loci were associated with eGFRcrea in the CHRIS study: here, we applied a one-side test and a Bonferroni correction of 0.05/147 (ie: the number of independent loci); this choice was made because the purpose of our analysis was to identify loci that could have been further characterized in the CHRIS study, despite its small sample size: further characterization would have implied assessment of mediation, interaction or other features; thus, here, the penalization was applied to subset loci with large effects. Please, see **Methods**, **page 6, lines 165-172**:

   "Given small-sample bias can cause large effect estimate fluctuations and given it is not uncommon to observe small studies with opposite effect directions compared to large GWAMA results, to ensure consistency of the effect direction, the association testing of the 147 loci in CHRIS was based on a one-sided test, following common practice [29]. Statistical significance was set at the Bonferroni-corrected level of 0.00034 (0.05/147), for any variant in strong LD (r2>0.8) with a lead CKDGen variant. LD was estimated using emeraLD v0.1 [30]. The total number of SNPs within the 147 loci was 6337. Adjustment for 147 independent tests covered 98% of the SNP's cumulative variance (S1 Fig in S1 File)."

   and **Discussion**, **pages 14-15, lines 439-447**:

   "Finally, our choice of adjustment for multiple testing is worth a brief reflection. We considered 147 loci that were already validated by at least one previous study through discovery and independent replication [17, 38, 58, 59]. As such, they wouldn't need further validation and a nominal significance level would be commonly accepted for hypothesis testing. However, the aim of our analysis was to identify loci with substantial effects in the CHRIS study to allow conducting meaningful mediation analyses. For this reason, we choose to penalize the significance level by the number of independent loci. This seems a sensible choice also by the fact that 147 independent tests covered 98% of the total variability of the 6337 SNPs in strong LD observed overall."

   (2) at the mediation analysis stage, to penalize for the number of tested mediators: please, see our **response to Reviewer 2, point 3**, which is about the same issue.

3. The use of a large and homogeneous sample, such as that of the CHRIS study, is a strength, as it allows for a more robust analysis of genetic associations. However, it is important to discuss the limitations of the methodology, such as the dependence on specific markers (such as creatinine) to estimate GFR and the need for validation in different populations.

Response: We fully agree with the Reviewer. We revised the Discussion of the strengths and limitations of our study for clarity. Please, see **Discussion**, **pages 14-15, lines 427-447**:

> "Our analysis has strengths and limitations. The analysis of a local homogeneous population sample was at the same time the most relevant strength and limitation of our investigation. While homogeneity may enhance the identification of specific genetic associations, it may also hinder the possibility to explain the underlying reasons of the observed larger effects compared to the average European ancestry population. The availability of a very large number of clinical parameters has allowed us to conduct an exhaustive and unbiased screening on potential mediation mechanisms. However, the analyzed traits lacked molecular specificity to identify underlying mechanisms. In addition to kidney function, eGFRcrea may partially reflect creatinine metabolism. Unfortunately, the CHRIS study did not measure alternative kidney function markers such as cystatin C or BUN. For this reason, we limited our analyses to loci whose kidney function relevance was already demonstrated by previous studies [17]. Assessing environmental exposures may also help explain the observed larger effects and would be ground for further research. Finally, our choice of adjustment for multiple testing is worth a brief reflection. We considered 147 loci that were already validated by at least one previous study through discovery and independent replication [17, 38, 58, 59]. As such, they wouldn't need further validation and a nominal significance level would be commonly accepted for hypothesis testing. However, the aim of our analysis was to identify loci with substantial effects in the CHRIS study to allow conducting meaningful mediation analyses. For this reason, we choose to penalize the significance level by the number of independent loci. This seems a sensible choice also by the fact that 147 independent tests covered 98% of the total variability of the 6337 SNPs in strong LD observed overall."

To help reasoning on the limitations of the methodology, we have now introduced a **new Figure 5**, which displays 6 possible scenarios that may originate the observed mediation effects, including collider bias and confounding by linkage disequilibrium. Please, see our Responses to **Reviewer 1, points 2 and 4**, and **Reviewer 2, point 4**.

The need for external validation is mentioned in several parts of the manuscript:

Abstract, **page 3, lines 52-53**:

> "The interplay between phenotypes at SHROOM3 and SLC34A1 and the role of thyroid function as a genetic effect modifier warrant further investigations."

We now added the following point to the **Discussion**, **page 14, lines 414-416**:

> "This result deserves replication in independent populations both to validate it and to assess the degree of generalizability of our results, which might be conditioned on specific environmental exposures." (see also **response to your point 5, below**)

and **Discussion**, **page 15, lines 454-456**:

> "Further investigations are warranted to link these findings to environmental and molecular characteristics of specific population samples …"

4. Results:

   It would be beneficial to include a section *discussing* the validation of the results in independent cohorts or the comparison with previous studies. This will help strengthen the credibility of the findings.

   Response: We fully agree with the Reviewer. For this reason, the need of validation of the results in independent cohorts is discussed extensively throughout the manuscript, in particular in the **Abstract** (last sentence), **Discussion** (**page 14, lines 414-416**), **Discussion** (**page 14, lines 418-420**), and **Conclusion** (last sentence, **page 15, lines 454-457**). Please, see response to previous point.

   The newly added evidence from eQTLs and pQTLs (**Results, page 10, lines 308-312**), along with discussion of the causal mechanisms (**new Figure 5**) further contextualize our findings.

Discussion:

The discussion about the limitations of the study should be more comprehensive. Consider discussing the homogeneity of the sample and how this may affect the generalizability of the results.

Response: We revised the strengths and limitation paragraph, in response to **point 3 from this Reviewer** (please, see above). In addition, we agree that the need of replication and assessment of generalizability should deserve more attention. For this reason, we added the following text to the **Discussion, page 14, lines 414-416**:

> "This result deserves replication in independent populations both to validate it and to assess the degree of generalizability of our results, which might be conditioned on specific environmental exposures."

We also corrected the reference to the sentence "STC1 is associated with urate and urea levels and is involved in the physiological response to dehydration and protein-rich diet" (**Discussion, page 14, lines 418-420**), which now refer more explicitly to evidence from a nomad Kenya population subjected to dehydration, where activation of the endocrine system seems to be the activator of STC1 causing kidney damage.

5. Conclusion:

   The conclusions are consistent with the results presented, but should emphasize the need for future investigations to explore the underlying mechanisms and applicability of the findings in clinical contexts.

   Response: In the last sentence of the **conclusions** we now state (**page 15, lines 454-457**):

   > "Further investigations are warranted to link these findings to environmental and molecular characteristics of specific population samples that may elucidate mechanisms not otherwise identifiable in large genetic meta-analyses."
